# Analysis of Energy Loss Characteristics of Vertical Axial Flow Pump Based on Entropy Production Method under Partial Conditions

**DOI:** 10.3390/e24091200

**Published:** 2022-08-27

**Authors:** Fan Yang, Pengcheng Chang, Yiping Cai, Zhikang Lin, Fangping Tang, Yuting Lv

**Affiliations:** 1 College of Hydraulic Science and Engineering, Yangzhou University, Yangzhou 225009, China; 2 Jiangxi Research Center on Hydraulic Structures, Jiangxi Academy of Water Science and Engineering, Nanchang 330029, China; 3 Hydrodynamic Engineering Laboratory of Jiangsu Province, Yangzhou University, Yangzhou 225009, China; 4 Water Resources Research Institute of Jiangsu Province, Nanjing 210017, China

**Keywords:** pump device, axial flow pump, conduit, energy loss, entropy production method, numerical analysis

## Abstract

The energy loss of the vertical axial flow pump device increases due to the unstable internal flow, which reduces the efficiency of the pump device and increases its energy consumption of the pump device. The research results of the flow loss characteristics of the total internal conduit are still unclear. Therefore, to show the internal energy loss mechanism of the axial flow pump, this paper used the entropy production method to calculate the energy loss of the total conduit of the pump device to clarify the internal energy loss mechanism of the pump device. The results show that the energy loss of the impeller is the largest under various flow conditions, accounting for more than 40% of the total energy loss of the pump device. The variation trend of the volume average entropy production and the energy loss is similar under various flow coefficients (*K*_Q_). The volume average entropy production rate (EPR) and the energy loss decrease first and then increase with the increase of flow, the minimum volume average entropy production is 378,000 W/m^3^ at *K*_Q_ = 0.52, and the area average EPR of the impeller increases gradually with the increase of flow. Under various flow coefficient *K*_Q_, the energy loss of campaniform inlet conduit is the smallest, accounting for less than 1% of the total energy loss. Its maximum value is 63.58 W. The energy loss of the guide vane and elbow increases with the increase of flow coefficient *K*_Q_, and the maximum ratio of energy loss to the total energy loss of the pump device is 29% and 21%, respectively, at small flow condition *K*_Q_ = 0.38. The energy loss of straight outlet conduit reduces first and then increases with the increase of flow coefficient *K*_Q_. When flow coefficient *K*_Q_ = 0.62, it accounts for 27% of the total energy loss of the pump device, but its area average entropy production rate (EPR) and volume average entropy production rate (EPR) are small. The main entropy production loss in the pump device is dominated by entropy production by turbulent dissipation (EPTD), and the proportion of entropy production by direct dissipation (EPDD) is the smallest.

## 1. Introduction

In the coastal plains of China, the large latitude span makes the climate difference between the north and the south, and the rainfall is obviously different in time and space, which makes the spatial and temporal distribution of water resources in China uneven. Therefore, the country vigorously constructs pumping stations to alleviate this problem. Axial flow pump is more and more extensively applied in low head pumping stations in coastal areas. From drainage pumping stations in plain areas to cross-basin water transfer projects in China, axial flow pump plays an irreplaceable role. In addition, axial flow pumps are also extensively applied as propulsion devices in the field of marine ships.

Axial flow pump devices can be classified as vertical pump devices, oblique pump devices and horizontal pump devices according to the arrangement form of the pump shaft [1]. A vertical pump device is also called a vertical axial flow pump device. It is extensively applied in cross-basin water transfer projects and urban municipal engineering in China [2]. Driven by the national dual carbon policy, it is required to optimize the vertical axial flow pump device to reduce hydraulic loss and improve efficiency, thereby reducing energy consumption. The traditional hydraulic loss method is calculated by the inlet and outlet pressure drop method. This method is relatively simple and fast, but it can only calculate the overall hydraulic loss of a certain section of the pump device and cannot be accurate to the local part of a certain overflow structure. Precise local structural optimization cannot be made in the structural optimization of the pump device. In order to visualize the hydraulic loss, relevant scholars introduced the entropy production method to calculate the fluid machinery to determine the location and amount of loss in the fluid machinery. For example, Gong et al. [3] used the entropy production method to quantitatively calculate the loss dissipation under the steady flow characteristics of the turbine and found that the dissipation of the guide vane accounted for 25% of the total device. Yu et al. [4] calculated the hydraulic loss of the Francis turbine under different mass flow rates based on entropy production theory and found that the energy loss of the draft tube of the Francis turbine was larger than that of other components. Huang et al. [5] calculated the entropy of ordinary centrifugal pumps and found that the direct dissipation entropy in the pump was very low, accounting for only 0.716%. EPTD and EPWS accounted for 54.629% and 44.654%, respectively, indicating that the influence of average velocity in the pump was small, and the main flow loss was caused by turbulent flow and wall shear stress. The entropy generation method is also applicable to quantify the energy loss in two-phase cavitation flow. Li et al. [6] studied the cavitation flow characteristics of NACA 0015 airfoil under various water temperatures and revealed the relationship between thermodynamic effect and energy loss of airfoil in cavitation based on the entropy production method. Ji et al. [7] analyzed the energy characteristics of the mixed flow pump under stall conditions by using CFD technology. By considering the relationship between turbulent dissipation, wall dissipation and blade thickness on total entropy production under stall conditions, the source of impeller domain loss was identified, which laid a foundation for the optimization of mixed flow pump. Gu et al. [8] studied the influence of the clock effect on the flow field and water loss in the pump when the pump as a turbine (PAT) and explored the change of internal energy loss of PAT under different guide vanes. Ghorani et al. [9] revealed the distribution of PAT hydraulic loss and found that the energy dissipation in the conduit accounted for more than 50%, and the main energy loss from EPTD accounted for 86.89–90.98%. Selecting a better turbulence model can more accurately simulate the internal flow pattern of fluid machinery. Li et al. [10] simulated the relationship between local steam flow distribution in centrifugal pumps and EPR distribution by using SST *k-ω* and Zwart models. Lai et al. [11] revealed that the turbulent dissipation of centrifugal pump cavitation mainly comes from eddy current, and the main source of entropy production by wall shear stress (EPWS) is the flow’s shear stress. In addition, the entropy production method is also applied to quantitatively calculate the energy dissipation in a non-aqueous fluid medium. Riaz et al. [12,13] studied the heat exchange and flow loss in Eyring–Powell fluid and nanofluid, revealing the flow heat transfer and viscous dissipation characteristics of Eyring–Powell fluid and nanofluid. Zhao et al. [14] studied the flow characteristics of the Ree–Eyring fluid mixed convection between two turntables; discussed the irreversibility of the fluid in the four states of heat generation/absorption, dissipation, radiation heat flux and Joule heating; and obtained the results consistent with the previous research results.

The entropy production method can visually calculate the energy dissipation of fluid machinery, which is convenient to observe the internal energy dissipation of fluid machinery. In recent years, relevant scholars introduced the entropy production method into the calculation of energy dissipation of axial flow pumps. Shen et al. [15] introduced the entropy production method to intuitively observe the mechanical energy dissipation loss of the tip clearance of the axial flow pump under various flow rates, indicating that the impeller’s energy dissipation has a strong correlation with the hump characteristics. Zhang et al. [16] analyzed the energy dissipation mechanism of axial flow pump stations under reverse power generation using by entropy production method. Yang et al. [17] calculated the internal flow energy loss of the inclined axial flow pump under some working conditions and found that the main flow loss was concentrated in the impeller region of the pump device. Fei et al. [18] studied the axial flow pump under cavitating operating conditions and calculated the local energy dissipation of the impeller chamber under cavitation conditions. It was found that the region with high vorticity was consistent with the region with high entropy production. Zhang et al. [19] analyzed the loss mechanism of the axial flow pump rotor and used two loss evaluation methods based on entropy production rate and the derivative of hysteresis enthalpy material to quantitatively evaluate energy dissipation. Li et al. [20] analyzed the influence of different blade root clearances on the mechanical energy dissipation of the pump rotor and found that the overall mechanical energy dissipation of the impeller increased with the increase of blade root clearance radius.

Domestic and foreign scholars conducted detailed studies on the energy dissipation of internal flow of fluid machinery such as centrifugal pumps [21,22], turbines [23,24] and axial flow pumps [25,26], but there are few studies on the energy loss characteristics of the total flow pipeline of the vertical axial flow pump device using the entropy production method. In this paper, the commercial software ANSYS CFX was used to conduct a three-dimensional (3D) numerical simulation of the pump device. Based on the entropy production method, the flow loss of the pump device under partial conditions was calculated, and the flow loss position and energy loss amount inside the pump device was revealed, which provides a reference for the optimization design of the structural details of the vertical axial flow pump device.

## 2. Mathematical Model and Numerical Simulation Method

### 2.1. Numerical Calculation Model

**Figure 1:**
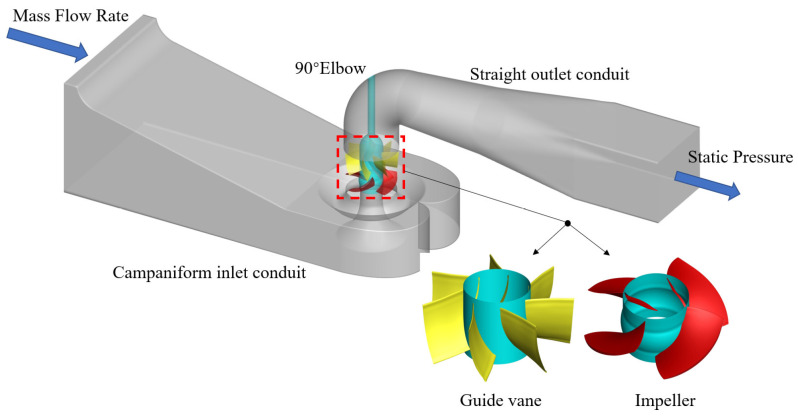
Three-dimensional model of vertical axial flow pump device.

The 3D model of the vertical axial flow pump device is shown in Figure 1. It is mainly composed of five flow components: campaniform inlet conduit, impeller, guide vane, 90° elbow and straight outlet conduit. The main geometric dimensions of each flow component and the main parameters of the pump device can be referred to in Ref. [27].

### 2.2. Numerical Method

In this paper, based on the ANSYS CFX solver, the finite volume method based on the finite element was used for the discretization of the control equation, and the discrete equation was solved by the fully implicit coupled algebraic multi-grid method. In the discretization process, the convection term adopts the high-resolution format, and the other terms adopt the central difference format [2]. The flow state of the pump device is intricate during operation, and the flow field changes significantly under various *K*_Q_. SST *k-ω* model integrates the benefits of *k-ω* and *k-ε* models, which can accurately simulate the flow separation of inverse pressure gradient and accurately predict the complex flow field distribution inside the pump device [28,29]. Therefore, the Reynolds-averaged N-S (Navier–Stokes) equation [30] and SST *k-ω* turbulence model were used to conduct a 3D numerical simulation of the flow field in the pump device. In the simulation, so as to refrain from the effect of initial speed distribution, the inlet extension part and outlet extension part were added before the campaniform inlet conduit and after the straight outlet conduit, respectively. The boundary conditions are set in Ref. [31], as shown in Table 1.

### 2.3. Grid Reliability Analysis

**Figure 2:**
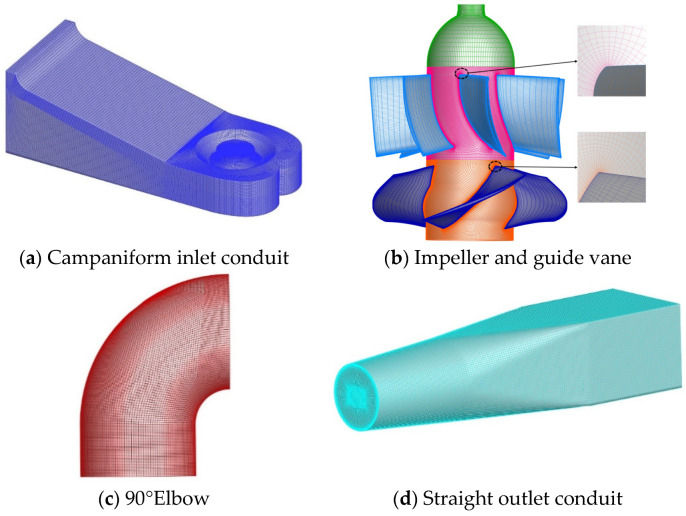
Grid diagram of each component.

ICEM CFD software was applied to conduct structured grid division for the flow conduit part and 90° elbow, and the grid quality of each flow component was above 0.4. ANSYS TURBOGRID software was applied to conduct structured grid division of the blade part. The grid of each component is shown in Figure 2.

In order to check the effect of grid number on numerical simulation accuracy, the grid number independence of the axial flow pump was analyzed when the *K*_Q_ = 0.52, as shown in Table 2. When the grid number outstrips 5.02 × 10^6^ and efficiency tends to stabilize, and the relative error is less than 0.2%, the influence of the number of grids on the numerical calculation can be ignored [32].

The grid reliability verification index uses GCI (Grid Convergence Index) as the evaluation standard [33,34]. Three groups of grids are selected from seven various grid numbers for grid convergence analysis. The three groups of grids are G1 = 2.2 million, G2 = 5.02 million, and G3 = 5.6 million, respectively. Based on the GCI calculation formula in Ref. [35], the results are shown in Table 3.

**Figure 3:**
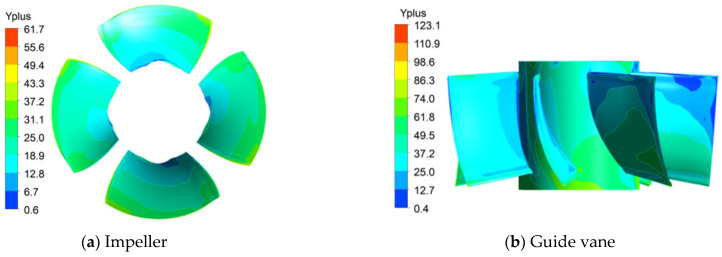

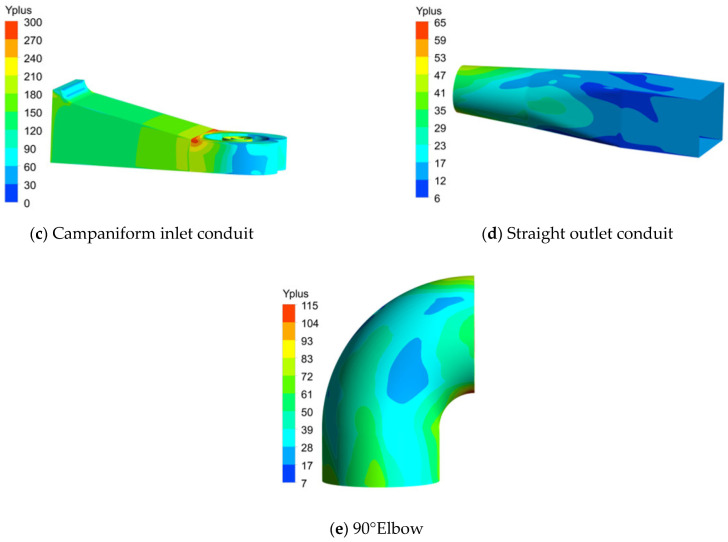
Wall Yplus cloud images of each component.

Both GCI_21_ and GCI_32_ are less than 1%, indicating a small discrete error [35]. Therefore, 5.02 × 10^6^ grids were finally used as the final numerical calculation of the grid number. The wall Yplus values of each component are shown in Figure 3, which meets the requirement of the wall Yplus value in Refs. [36,37].

### 2.4. Entropy Production Method

The entropy production method is derived from the second law of thermodynamics. By calculating the entropy production of the flow components of the pump device, the unstable region of the flow can be intuitively characterized. The entropy production forms that quantitatively describe the energy loss in the pump device can be divided into four types, which are induced by the time-averaged velocity, the pulsating velocity, the average temperature gradient and the pulsating temperature gradient. When the pump device works in the pure water medium, the proportion of entropy production caused by temperature change is small, and the internal heat exchange can be ignored. Therefore, the flow entropy production of the pump device during operation only considers the entropy production by direct dissipation (EPDD) and the entropy production by turbulent dissipation (EPTD). In addition, the pump device wall has a high-velocity gradient, so there is a large energy dissipation at the wall. Therefore, the entropy production by wall shear stress (EPWS) should be considered when studying the entropy production loss of the pump device.

Equation (1) was used to solve EPDD.

(1) $$\overset{\cdot }{S_{\bar{D}}^{\prime \prime \prime }}=\frac{2\mu_{eff}}{T}\left[{\left(\frac{\partial \bar{u_{1}}}{\partial x_{1}}\right)}^{2}+{\left(\frac{\partial \bar{u_{2}}}{\partial x_{2}}\right)}^{2}+{\left(\frac{\partial \bar{u_{3}}}{\partial x_{3}}\right)}^{2}\right]+\frac{\mu_{eff}}{T}\left[{\left(\frac{\partial \bar{u_{2}}}{\partial x_{1}}+\frac{\partial \bar{u_{1}}}{\partial x_{2}}\right)}^{2}+{\left(\frac{\partial \bar{u_{2}}}{\partial x_{3}}+\frac{\partial \bar{u_{3}}}{\partial x_{2}}\right)}^{2}+{\left(\frac{\partial \bar{u_{3}}}{\partial x_{1}}+\frac{\partial \bar{u_{1}}}{\partial x_{3}}\right)}^{2}\right]$$

(2) $$\overset{\cdot }{S_{D^{\prime }}^{\prime \prime \prime }}=\frac{2\mu_{eff}}{T}\left[{\left(\frac{\partial u_{1}^{\prime }}{\partial x_{1}}\right)}^{2}+{\left(\frac{\partial u_{2}^{\prime }}{\partial x_{2}}\right)}^{2}+{\left(\frac{\partial u_{3}^{\prime }}{\partial x_{3}}\right)}^{2}\right]+\frac{\mu_{eff}}{T}\left[{\left(\frac{\partial u_{2}^{\prime }}{\partial x_{1}}+\frac{\partial u_{1}^{\prime }}{\partial x_{2}}\right)}^{2}+{\left(\frac{\partial u_{2}^{\prime }}{\partial x_{3}}+\frac{\partial u_{3}^{\prime }}{\partial x_{2}}\right)}^{2}+{\left(\frac{\partial u_{3}^{\prime }}{\partial x_{1}}+\frac{\partial u_{1}^{\prime }}{\partial x_{3}}\right)}^{2}\right]$$

In the SST *k-ω* model, the local entropy production loss induced by velocity fluctuation is closely related to *ω* [38,39]. Therefore, EPTD can be solved by Equation (3).

(3) $$\overset{\cdot }{S_{D^{\prime }}^{\prime \prime \prime }}=\beta \frac{\rho \omega k}{T}(\beta =0.09)$$

Equation (4) was used to solve EPWS.

(4) $$\overset{\cdot }{S_{w}^{\prime \prime \prime }}=\frac{\vec{\tau }\cdot \vec{v}}{T}$$

For the entropy production calculation of different computational domains, the total entropy production loss of the computational part can be acquired by integrating the volume of the local entropy production. The EPWS can be integrated by the area of the calculation domain.

(5) $$S_{pro,\bar{D}}=\int_{v}\overset{\cdot }{S_{\bar{D}}^{\prime \prime \prime }}dV$$

(6) $$S_{pro,D^{\prime }}=\int_{v}\overset{\cdot }{S_{D^{\prime }}^{\prime \prime \prime }}dV$$

(7) $$S_{pro,W}=\int_{A}\frac{\vec{\tau }\cdot \vec{v}}{T}dA$$

(8) $$S_{pro}=S_{pro,\bar{D}}+S_{pro,D^{\prime }}+S_{pro,W}$$

(9) $$P=T\cdot S_{pro}$$

where $\mu_{eff}$ is effective dynamic viscosity, ω is turbulent eddy viscosity frequency, *k* is turbulent kinetic energy, $\vec{\tau }$ is wall shear stress, $\vec{v}$ is the velocity vector of the first grid center at the wall, $S_{pro,\bar{D}}$ is EPDD, $S_{pro,D^{\prime }}$ is EPTD, $S_{pro,W}$ is EPWS, *V* is fluid volume, *A* is the wall area and *P* is energy loss.

### 2.5. Reliability Verification of Numerical Calculation and Entropy Production Calculation

**Figure 4:**
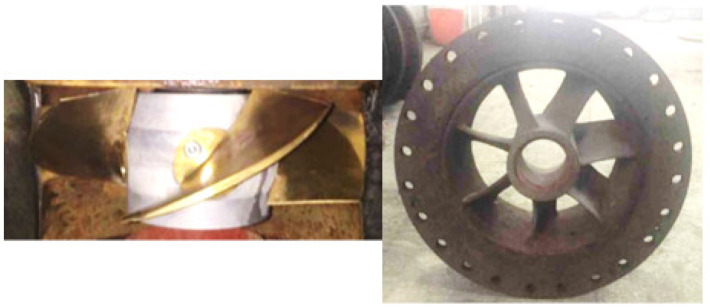
Physical models of impeller and guide vane.

**Figure 5:**
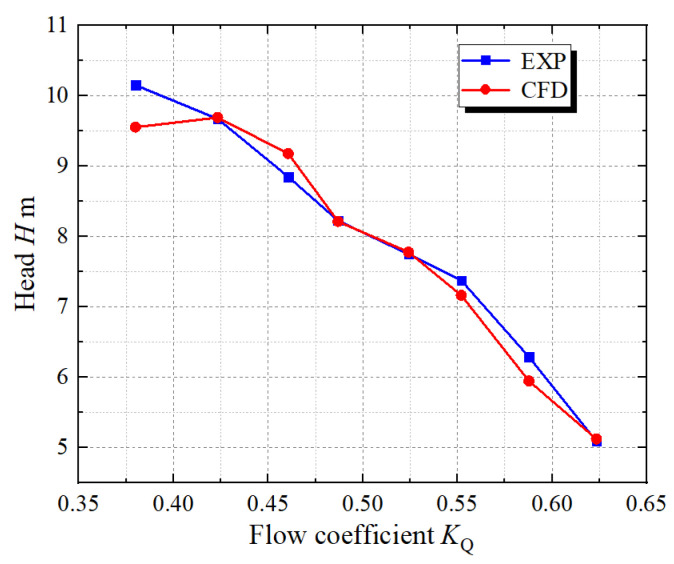
Head comparison between CFD and test.

The physical model test of the vertical axial flow pump device is conducted on the high-precision hydraulic and mechanical test bench of the Hydrodynamic Engineering Laboratory of Jiangsu Province, China. The optimal flow condition of the physical model of the pump device is 338 L/s, and the rotational speed is 1433 r/min. The nominal impeller diameter of the model pump is *D* = 300 mm, the hub ratio is 0.483, and it has 4 blades, which are formed by numerical control machining of brass material. The hub diameter of the guide vane is 140 mm, and it has 7 blades; its main material is steel. The physical models of the blade part are shown in Figure 4. The inlet and outlet conduits are welded with steel plates. The head of the test pump device is measured by the EJA110A differential pressure transmitter, and the transfer torque of the model pump shaft is directly measured by the ZJ torque meter. The flow rate is measured by E-mag electromagnetic flowmeter. The pressure differential transmitter, torque meter and flowmeter are calibrated by national measurement units [40]. In Ref. [41], the flow coefficient *K*_Q_ is introduced, and the formula is shown in Equation (10). The head comparison between experimental and CFD is shown in Figure 5. The maximum relative error of the head is 5.44% under *K*_Q_ = 0.38, and the relative error of the head is less than 4% under other conditions, and the data match well, indicating that the head predicted by CFD is more accurate.

(10) $$K_{Q}=\frac{Q}{nD^{3}}$$

(11) $$\Delta H_{\mathrm{EPR}}=\frac{S_{pro}\cdot T}{\overset{•}{m}g}$$

(12) $$\eta_{\mathrm{EPR}}=\frac{H-\Delta H_{\mathrm{EPR}}}{H}\times 100\%$$

(13) $$\gamma_{\Delta p-\mathrm{EXP}}=\frac{\left.\left|\eta_{\mathrm{EXP}}-\eta_{\Delta p}\right.\right|}{\eta_{\mathrm{EXP}}}\times 100\%$$

(14) $$\gamma_{\Delta p-\mathrm{EPR}}=\frac{\left.\left|\eta_{\Delta p}-\eta_{\mathrm{EPR}}\right.\right|}{\eta_{\Delta p}}\times 100\%$$

where *K*_Q_ is flow coefficient; *Q* is the flow rate of the pump device, which are, respectively, 245.63 L/s, 273.33 L/s, 297.59 L/s, 338.22 L/s, 371.06 L/s and 402.29 L/s; *n* is the rotational speed, which is 1433 r/min; *D* is impeller diameter; △*H*_EPR_ is entropy production head loss; *η*_EPR_ is entropy production efficiency; $\overset{•}{m}$ is mass flow rate; *g* is 9.81 m/s^2^; *T* is temperature; *H* is the head; *γ*_△p-EXP_ is the relative error between CFD efficiency and experimental efficiency; *γ*_△p-EPR_ is the relative error between CFD efficiency and entropy production efficiency; *η*_△p_ is the numerical simulation of pump efficiency; and *η*_EXP_ is the test pump efficiency.

**Figure 6:**
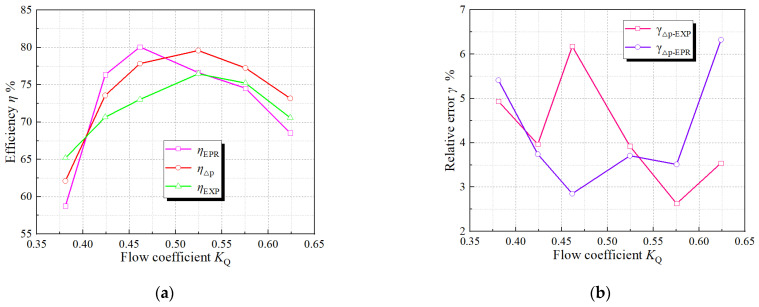
Comparison of pump device efficiency among pressure drop method, entropy production method and test. (**a**) Efficiency comparison, (**b**) Relative error.

The operation efficiency is a target to measure the comprehensive operation of the axial flow pump device. In Ref. [4], the entropy production head loss and entropy production loss efficiency are introduced, and they are solved by Equations (11) and (12). Equation (13) defines the relative error between the total pressure efficiency and the test efficiency, and Equation (14) defines the relative error between the total pressure efficiency and the entropy production efficiency. Figure 6 shows the comparison of entropy production efficiency *η*_EPR_, experimental efficiency and CFD efficiency. The efficiency error of CFD and experiment and the relative error of numerical simulation total pressure efficiency and entropy production efficiency are shown in Figure 6b. As shown in Figure 6, the variation trends of entropy production efficiency, total pressure efficiency and test efficiency under various *K*_Q_ are consistent, and the relative error *γ* is below 6.5%, indicating that the reliability of numerical simulation and entropy production calculation is high [42].

## 3. Results and Analysis

**Figure 7:**
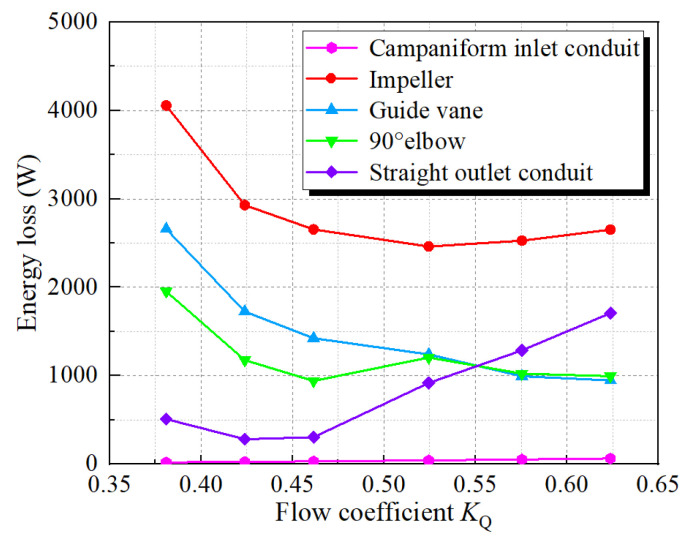
Energy loss of each component under various *K*_Q._

**Figure 8:**
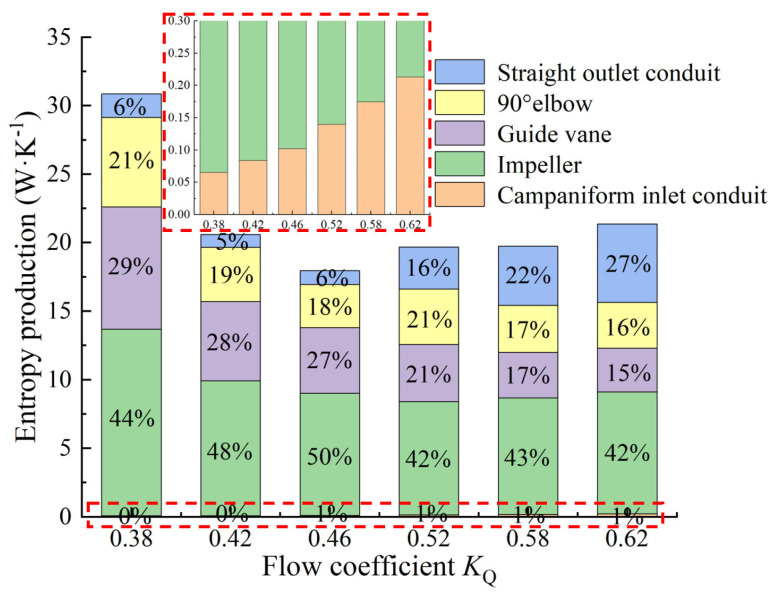
Proportion of entropy production loss of each flow component under various *K*_Q._

**Figure 9:**
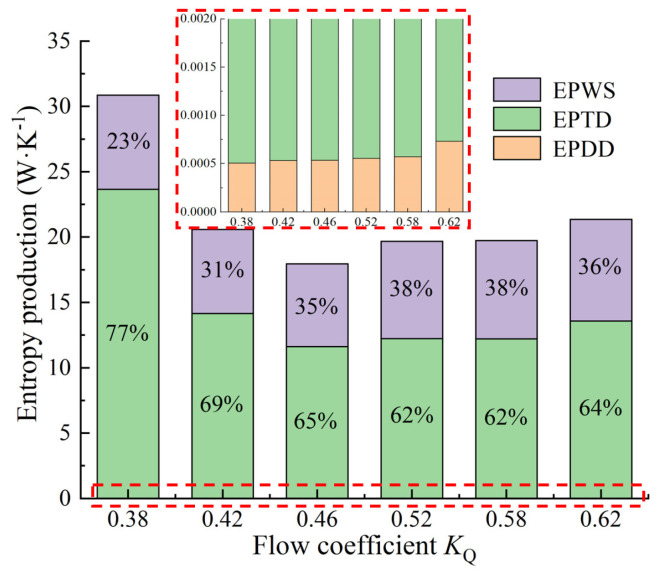
Proportions of EPDD, EPTD and EPWS under various *K*_Q._

Figure 7 shows the energy loss of the flow components under various *K*_Q_. The ratio of the entropy production loss of each flow component to the total entropy production loss is shown in Figure 8. The energy loss of the impeller is the largest among the flow components, and the proportion under various *K*_Q_ is more than 40%, with the maximum value exceeding 4000 W. When *K*_Q_ = 0.52, the energy loss is the smallest, and it reduces first and then increases from *K*_Q_ = 0.38 to *K*_Q_ = 0.62. The energy loss of the campaniform inlet conduit is the smallest, and the energy loss accounts for less than 1%, indicating that the campaniform inlet conduit has good hydraulic performance and a good guiding effect on the flow so that the flow enters the impeller smoothly. The maximum is 63.58 W at *K*_Q_ = 0.62. The energy loss of the guide vane is the second, but under *K*_Q_ = 0.58 and *K*_Q_ = 0.62, the energy loss of the straight outlet conduit exceeds the energy loss of the guide vane. The variation trend of energy loss of elbow is consistent with that of impeller and guide vane, but when *K*_Q_ = 0.52–0.62, the energy loss of elbow and guide vane is very close. The total entropy production loss of the pump device is the largest when *K*_Q_ = 0.38. With the increase of *K*_Q_, the total entropy production loss of the pump device reduces first and then increases. Figure 9 shows the proportions of EPDD, EPTD and EPWS under various *K*_Q_. The proportion of EPTD is above 60% under different *K*_Q_. With the increase in *K*_Q_, the proportion of EPTD shows a downward inclination. EPDD has the smallest proportion, which is less than 1% under various *K*_Q_ and has an ascending inclination with the increase of *K*_Q_. The variation inclination of EPWS proportion is similar to that of EPDD, and the largest proportion of EPWS is 38% when *K*_Q_ = 0.52 and *K*_Q_ = 0.58. EPWS and EPTD reduce first and then increase from *K*_Q_ = 0.38 to *K*_Q_ = 0.62. The EPTD is caused by the turbulence of the water flow. The flow state in the pump is better when *K*_Q_ = 0.46 and *K*_Q_ = 0.52 near the optimal working condition, so the EPTD is small. EPWS is caused by the shear stress generated by the water flow on the wall of the pump device. When the flow rate increases, the velocity gradient of the water flow compared with the wall surface increases, so the increase of the shear stress generated by the water flow at the wall surface leads to the increase of EPWS.

Figure 6 illustrates the total energy loss of each flow component under different flow coefficients, but each flow component has different volumes and surface areas. Therefore, the total energy loss cannot clarify the contribution of each flow component to the entropy production. Therefore, in order to reveal the distribution of entropy production rate per unit area and volume, in Ref. [4], the volume average entropy production and area average entropy production were introduced, and the calculation formulas are shown in Equations (15)–(18). Volume average EPR is the average value of EPDD and EPTD on the unit volume of each flow passage component, and its unit is W/m^3^. The area average entropy production is the average value of the wall entropy production on the unit area of each flow passage component, and its unit is W/m^2^.

(15) $$P_{\bar{D},V}=\frac{T\cdot S_{pro,\bar{D}}}{V}$$

(16) $$P_{D^{\prime },V}=\frac{T\cdot S_{pro,D^{\prime }}}{V}$$

(17) $$P_{V}=P_{D^{\prime },V}+P_{\bar{D},V}$$

(18) $$P_{W,A}=\frac{T\cdot S_{pro,W}}{A}$$

where *P**_V_*** is volume average EPR, and *P_W_*_,*A*_ is the area average EPR.

**Figure 10:**
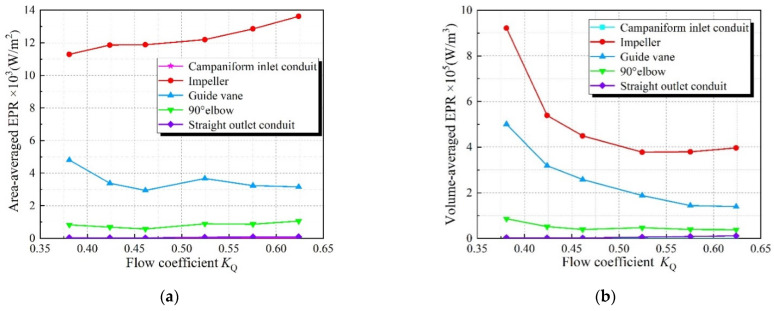
Volume average EPR distribution and area average EPR distribution of each flow passage component. (**a**) Area average EPR, (**b**) Volume average EPR.

Figure 10 shows the volume average EPR distribution and area average EPR distribution of each flow structure. Under various *K*_Q_, the volume average EPR and area average EPR of the impeller are the largest, the volume average EPR is higher than 400,000 W/m^3^, and the area average EPR is higher than 11,000 W/m^2^. With the increase of *K*_Q_, the area average EPR increases while the volume average EPR decreases. This is because when the rotational speed is constant, the larger the *K*_Q_ is, the larger the shear stress generated by the wall, and the flow causes the increase of the area average EPR, and the increase of the *K*_Q_ reduces the pressure difference on the rotor surface, resulting in the decrease of the tip clearance leakage and the decrease of the volume average EPR of the impeller. The area average EPR and volume average EPR of the guide vane is in the second place, and the energy loss is larger when the *K*_Q_ is small. This is because the complex surface of the guide vane and the change of the vortex inside the guide vane lead to large energy loss in the unit volume and unit area of the guide vane. Compared with the impeller and guide vane, the volume average EPR and area average EPR of other flow components is much smaller, the volume average EPR is less than 100,000 W/m^3^, and the area average EPR is less than 1100 W/m^2^. The volume average EPR and area average EPR of the elbow has little change, and the change law is similar to that of the impeller. The volume average EPR and area average EPR of campaniform inlet conduit increases with the increase of *K*_Q_. The volume average EPR and area average EPR of straight outlet conduit decrease first and then increase with the increase of *K*_Q_. Overall, the energy loss of the straight pipe outlet is large, but the entropy yield per unit area and volume is small.

**Figure 11:**
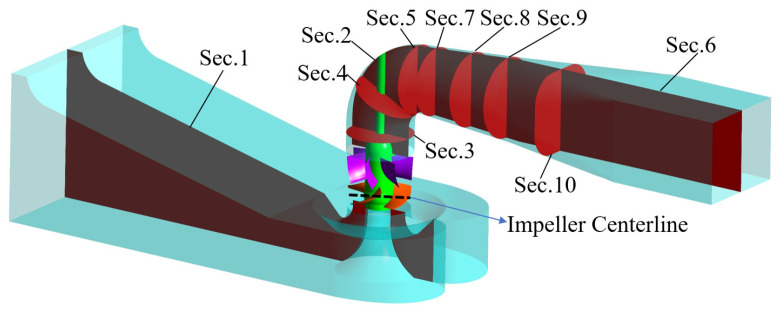
Diagram of characteristic sections of vertical axial flow pump device.

Ten typical sections are chosen inside the pump device to facilitate the display of the EPR of the whole conduit. The distribution of each section is shown in Figure 11. Sec.1, Sec.2 and Sec.6 are located at the center of the flow pipeline, respectively. Sec.3 and Sec.4 are located at 1.0 *D* and 1.667 *D* from the impeller center line, and Sec.5, Sec.7, Sec.8 and Sec.9 are located at 0.833 *D*, 1.0 *D*, 1.667 *D*, 2.333 *D* and 3.333 *D* from the pump shaft.

**Figure 12:**
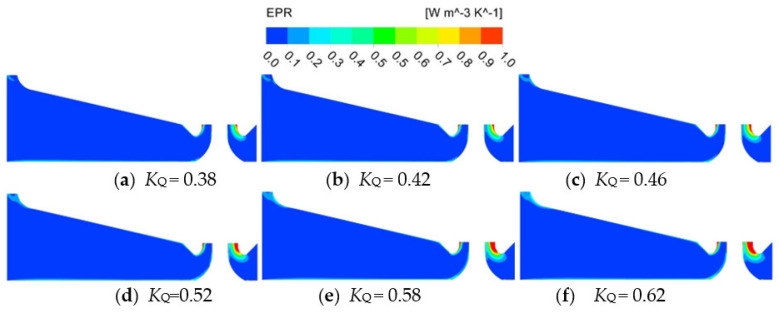
EPR distribution in characteristic section of campaniform inlet conduit.

Figure 12 shows the EPR of campaniform inlet conduit Sec.1. The energy loss in the campaniform inlet conduit is the smallest (Figure 7), mainly concentrated on the right side of the outlet diversion cone of the conduit, and the loss in other areas is small, indicating that the internal flow of the campaniform inlet conduit is good. At *K*_Q_ = 0.38, the area with large energy loss at the campaniform inlet conduit outlet is small, and the area gradually increases with the increase of *K*_Q_. With the increase of flow coefficient, the flow velocity inside the campaniform inlet conduit increases, and the velocity gradient of the flow relative to the wall increases, resulting in the increase of the shear stress of the flow on the wall. Under the guidance of the guide cone, the flow shrinks at the conduit outlet, and the velocity changes greatly at the gradual contraction conduit outlet, resulting in a large flow energy loss. This situation is most obvious in the case of large flow, which is consistent with the change of the energy loss of the companiform inlet conduit shown in Figure 7.

**Figure 13:**
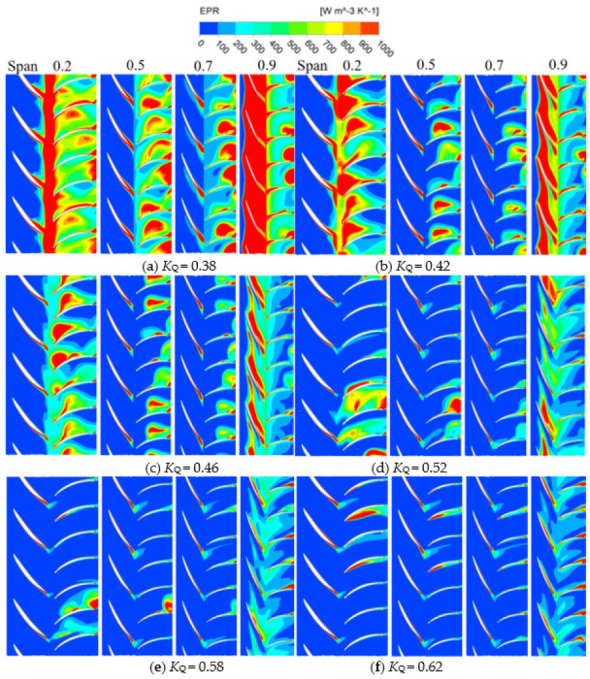
EPR distribution in various spanwise of guide vane and impeller.

In the pump device, the impeller, as a core part, converts mechanical energy into kinetic energy of water, and its conversion process produces irreversible energy loss. The guide vane improves the flow pattern, and there is also a large amount of energy loss in the recovery of water circulation. Figure 13 shows the EPR distribution map of various span impellers and guide vane. The larger EPR occurs at Span = 0.2 and Span = 0.9, and the main energy loss comes from the impeller, which is concentrated in the impeller and the guide vane inlet. These losses are most obvious when *K*_Q_ = 0.38 and *K*_Q_ = 0.42. The distribution of high EPR is mainly concentrated in Span = 0.9 and Span = 0.2, Span = 0.9, close to the tip clearance. Under small flow conditions, the tip clearance discharge increases [37,38,39], and Span = 0.2 is close to the hub. This is affected by the airfoil structure of the impeller blade, which is prone to deflow and other phenomena, resulting in the increase of rotor mechanical energy dissipation. When *K*_Q_ = 0.38, the high EPR areas of Span = 0.5 and Span = 0.7 are mainly distributed at the trailing edge of the impeller blade and the back of the guide blade. This is mainly due to the phenomenon of flow separation at the trailing edge of the impeller blade, and the intensification of flow separation under the condition of a small flow rate increases the energy loss [16]. The energy loss inside the guide vane mainly comes from the eddy current inside the guide vane. With the increase of flow rate, the flow pattern inside the guide vane becomes better [27], and the energy loss decreases with the decrease of eddy current. With the increase of *K*_Q_, the EPR inside the guide vane moves to the trailing edge of the guide vane, and the EPR reduces, which is consistent with the change of energy loss shown in Figure 7.

**Figure 14:**
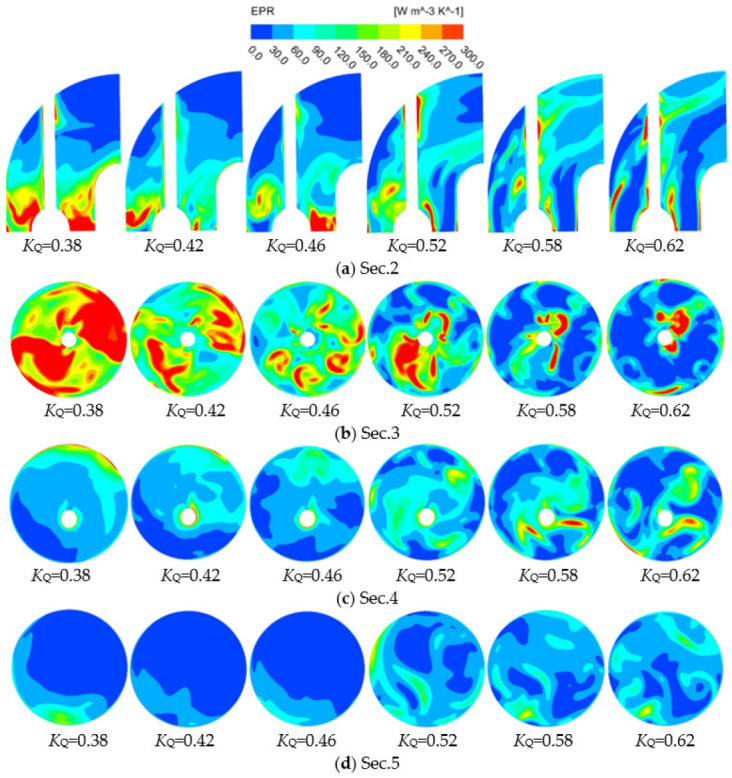
EPR distribution of elbow sections.

Figure 14 shows the EPR distribution cloud chart of the 90° elbow section under various *K*_Q_. When *K*_Q_ = 0.38–0.46, the EPR distribution is mainly concentrated near the elbow inlet guide cap, and the EPR distribution at the outlet is small. As shown in Figure 14b, the EPR at the elbow inlet Sec.3 decreases with the increase of *K*_Q_. When the flow is large, the flow needs to complete the 90° turning at the elbow, and the elbow outlet is susceptible to the change of flow in the pipeline. Therefore, the EPR at the elbow turning is significantly increased compared with that under *K*_Q_ = 0.38. Therefore, when *K*_Q_ = 0.52–0.62, the EPR distribution of the elbow is mainly focused on the turning point and transfers to the elbow outlet. In Sec.3–Sec.5, the EPR distribution is asymmetric under various *K*_Q_, which is related to the uneven flow distribution at the guide vane outlet and the uneven flow distribution caused by the residual circulation at the guide vane outlet [27].

**Figure 15:**
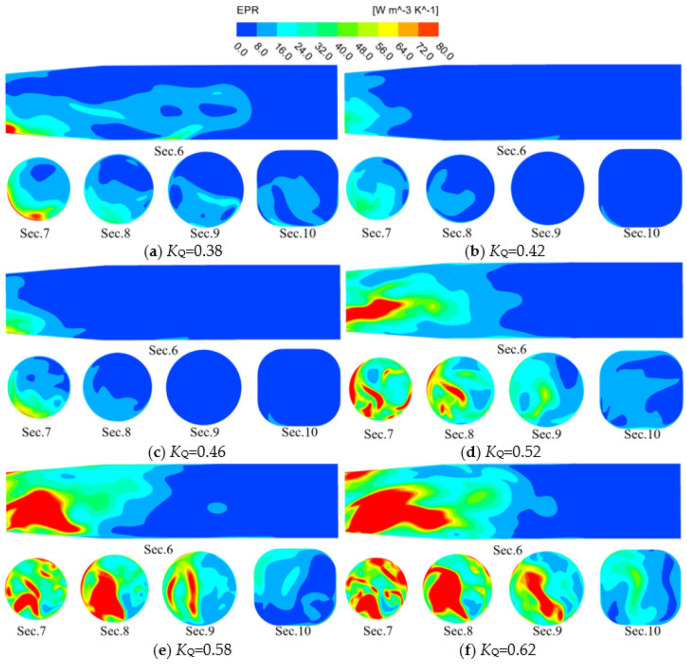
EPR distribution in characteristic sections of straight outlet conduit.

Figure 15 shows the EPR distribution of the straight outlet conduit characteristic section under various *K*_Q_. The EPR distribution of the straight outlet conduit is mainly focused on the linear shrinking section of the conduit, and with the increase of *K*_Q_, the distribution area of high EPR gradually increases. The high EPR distribution area is the smallest when *K*_Q_ = 0.42, and the high EPR distribution area is the largest when *K*_Q_ = 0.62, which is consistent with the changing trend of total energy loss of straight outlet conduit shown in Figure 7. When *K*_Q_ = 0.38, the EPR is mainly distributed in the middle and lower sections of the flow conduit. When *K*_Q_ = 0.42 and 0.46, the EPR is mainly distributed near the flow pipeline inlet. It can be seen from the EPR cloud images of Sec. 7 and Sec. 8. At this time, the EPR is mainly concentrated in the flow pipeline lower left. When *K*_Q_ = 0.52–0.62, the high EPR distribution area extends from the flow pipeline inlet to the interior. As shown in the cloud image of Sec. 6–Sec. 10, the high EPR distribution area is mainly concentrated in the flow pipeline’s lower half and concentrated on the flow pipeline’s left side, which is related to the phenomenon of partial flow in the pump device outlet conduit shown in Ref. [40], which is mainly caused by the residual circulation of water.

## 4. Conclusions

In this article, the entropy production method was used to visualize the internal energy loss of the vertical axial flow pump device; the reliability of entropy production calculation was checked by comparing the total pressure efficiency, entropy production efficiency and test efficiency. The energy loss characteristics of the total flow conduit of the vertical axial flow pump device were studied, and the article drew three conclusions:

1. The energy loss of the impeller is the largest in the vertical axial flow pump device. From *K*_Q_ = 0.38 to *K*_Q_ = 0.62, the energy loss of the impeller reduces first and then increases, and the volume average EPR is consistent with the energy loss. The area average EPR is minimum at *K*_Q_ = 0.38 and maximum at *K*_Q_ = 0.62. The high EPR of the impeller is mainly focused on the rim, which is influenced by the tip clearance leakage. The energy loss of the guide vane reduces as the *K*_Q_ increases. The high EPR of the guide vane is mainly distributed on the backside and trailing edge of the vane;
2. The energy loss of the campaniform inlet conduit is the smallest, and the ratio of energy loss to total energy loss is less than 1% under various *K*_Q_. The high EPR distribution area is mainly concentrated in the outlet area. The volume average EPR and area average EPR of campaniform inlet conduit increase with the increase of *K*_Q_. The energy loss trend of the elbow is similar to that of the guide vane, which reduces with the increase of *K*_Q_. From *K*_Q_ = 0.38 to *K*_Q_ = 0.62, the high EPR area develops from the inlet guide cap to the elbow outlet. When *K*_Q_ = 0.62, the maximum energy loss of the straight outlet conduit is 1707 W, and when *K*_Q_ = 0.42, the minimum is 281 W. The energy loss, volume average EPR and area average EPR all decrease first and then increase with the increase of *K*_Q_. The main loss is distributed in the linear shrinking section of the conduit;
3. The energy dissipation of the flow in the pump device is mainly turbulent dissipation. Under various flow rates, the EPTD accounts for the largest proportion, up to 77%. The EPDD accounted for less than 1%, and the EPWS increased by 15% when *K*_Q_ = 0.52 and *K*_Q_ = 0.58. With the increase of *K*_Q_, the EPWS decreased first and then increased.

The vertical axial flow pump device has high efficiency in operation (but there is still unstable flow within it during operation) in order to pursue a more efficient axial flow pump device. In this paper, the entropy production method was used to determine the specific position of energy loss and the main causes of loss, and then the optimization measures can be carried out accordingly. This paper can provide a reference for the optimization and design of the hydraulic structure of vertical axial flow pump devices with higher efficiency. This is the author’s next stage of work.

## Figures and Tables

**Table 1:** Boundary condition settings.

Position	Boundary Types
Inlet surface of water inlet extension part	Mass flow rate
Exit surface of water outlet extension part	Standard atmospheric pressure
Pump device wall	No-slip wall
Interface on both sides of impeller	Frozen Rotor
Other computing domain interfaces	None
Convergence precision	10^−5^

**Table 2:** Analysis of grid independence.

Grid Scheme	Grid Number/10^4^	Efficiency/%	Relative Error/%
1	220	85.79	
2	284	84.68	−1.31
3	330	82.50	−2.69
4	382	81.67	−0.97
5	426	79.74	−2.42
6	502	79.59	−0.18
7	560	79.58	−0.02

**Table 3:** Grid convergence index.

Grid Number/10^4^	*r*	*p* *	*f*	*ε*	GCI/%
220			85.789		
502	1.316519	3.2689	79.5911	−0.07787	GCI_21_ = 0.0668
560	1.037118	3.2689	79.5764	−0.00018	GCI_32_ = 0.0018

*p* * is the convergence order in the grid convergence index.

## Data Availability

All data necessary to carry out the work in this paper are included in the figures and tables or are available in cited references.
